# Esterase LpEst1 from *Lactobacillus plantarum*: A Novel and Atypical Member of the αβ Hydrolase Superfamily of Enzymes

**DOI:** 10.1371/journal.pone.0092257

**Published:** 2014-03-24

**Authors:** Yanaisis Alvarez, María Esteban-Torres, Álvaro Cortés-Cabrera, Federico Gago, Iván Acebrón, Rocío Benavente, Karin Mardo, Blanca de las Rivas, Rosario Muñoz, José M. Mancheño

**Affiliations:** 1 Department of Crystallography and Structural Biology, Institute of Physical Chemistry Rocasolano, CSIC, Madrid, Spain; 2 Center of Advanced Studies of Cuba, CITMA, Havana, Cuba; 3 Laboratory of Bacterial Biotechnology, Institute of Food Science and Technology and Nutrition (ICTAN), CSIC, Madrid, Spain; 4 Department of Biomedical Sciences, School of Medicine and Health Sciences, University of Alcalá, Alcalá de Henares, Madrid, Spain; 5 Institute of Molecular and Cell Biology, University of Tartu, Tartu, Estonia; University Paris Diderot-Paris 7, France

## Abstract

The genome of the lactic acid bacterium *Lactobacillus plantarum* WCFS1 reveals the presence of a rich repertoire of esterases and lipases highlighting their important role in cellular metabolism. Among them is the carboxylesterase LpEst1 a bacterial enzyme related to the mammalian hormone-sensitive lipase, which is known to play a central role in energy homeostasis. In this study, the crystal structure of LpEst1 has been determined at 2.05 Å resolution; it exhibits an αβ-hydrolase fold, consisting of a central β-sheet surrounded by α-helices, endowed with novel topological features. The structure reveals a dimeric assembly not comparable with any other enzyme from the bacterial hormone-sensitive lipase family, probably echoing the specific structural features of the participating subunits. Biophysical studies including analytical gel filtration and ultracentrifugation support the dimeric nature of LpEst1. Structural and mutational analyses of the substrate-binding pocket and active site together with biochemical studies provided insights for understanding the substrate profile of LpEst1 and suggested for the first time the conserved Asp173, which is adjacent to the nucleophile, as a key element in the stabilization of the loop where the oxyanion hole resides.

## Introduction

Hydrolases constitute a class of enzymes that catalyse the hydrolysis of a wide variety of substrates, from peptides, amides or halides in addition to esters and triglycerides, as well as non-natural substrates. Although this assortment of substrates has complicated their classification, *de facto* they have been typified according to their known specificity. Esterases (EC 3.1.1), for instance, were defined as enzymes that hydrolyse ester bonds. Characteristically, they show specificity for either the alcohol or the acid moiety of the substrate, but not for both. Carboxylesterases (EC 3.1.1.1), in particular, catalyses the hydrolysis of small carboxylic acid ester-containing molecules at least partially soluble in water, while lipases (EC 3.1.1.3) display maximal activity against water-insoluble long-chain triglycerides [1]. Thus, although catalytically similar, lipases and carboxylesterases must deal with physicochemically distinct environments: whereas lipases have to be capable of identifying an insoluble or heavily aggregated substrate, i.e. a water-substrate interface [2], [3], carboxylesterase activity is maximal against monomeric substrates. Within this latter group of carboxylesterases substrate specificities vary widely, with some enzymes displaying highly specific activity towards particular esters such as acetylcholinesterase [4], heroin esterase [5] or Brefeldin A esterase [6], whereas others have activity against a broad range of substrates [7]. This group of enzymes is attractive for industry and in fact many carboxylesterases have been utilized in the synthesis of ester compounds in non-aqueous solvents and also in stereospecific hydrolysis since they combine a broad specificity range with a high stereoselectivity [8]–[10].

The ESTHER database of lipases and esterases classifies these enzymes into four blocks, C, H, L and X [11] according to their amino acid sequence. Block H includes the hormone-sensitive lipase (HSL) family, a group of lipases and carboxylesterases from diverse biological sources which share sequence similarities with mammalian HSL [12]. Apart from the characteristic GXSXG motif around the active site serine, which is also found in serine proteases [13], they contain a highly conserved sequence of HGGG upstream the catalytic site.

From a structural viewpoint, the amino acid sequence data on carboxylesterases indicate that they belong to the αβhydrolase superfamily of enzymes [14]–[16]. Members of this superfamily share a characteristic αβ fold, which is based on an eight-stranded mostly parallel βsheet surrounded on both sides by α-helices. This structural framework supports a catalytic machinery based on a catalytic triad made up of a nucleophile, usually serine, an acid (Asp/Glu) and a histidine. The nucleophile is located within the above mentioned conserved G-X-S-X-G motif in a sharp turn between strand β5and helix α3called the nucleophile elbow, where it can be approached by the substrate and by the hydrolytic water molecule [16]. The specific structural arrangement of the active site, particularly the geometry of the nucleophile elbow and the connecting loop in which the HGGG motif is located, contribute to the formation of the so-called oxyanion hole, usually formed by two or three backbone nitrogen atoms, which is needed to stabilize the negatively charged transition state [17].

Recently, crystal structures of several bacterial HSL enzymes have been reported, including carboxylesterases PestE and AFEST from the archaea *Pyrobaculum calidifontis* [18] and *Archaeoglobus fulgidus* [19], respectively, carboxylesterases from environmental samples EstE7 (PDB entry: 3k6k) EstE5 [20] and Est25 [21], esterases EstE2 and Sto-Est from *Alicyclobacillus acidocaldarius* [22] and *Sulfolobus tokodaii* [23], heroin esterase from *Rhodococcus* sp. [5], Brefeldin A esterase (BFAE) from *Bacillus subtilis* [6] and carboxylesterase Cest-2923 from *Lactobacillus plantarum* WCFS1 [24]. A notable source of structural variability of this group of αβ hydrolase enzymes emerges from the diverse quaternary structures observed: from monomers like EstE5 [20] to octamers identified within the crystals of AFEST [19]. These findings reveal the remarkable versatility of the αβ hydrolase fold in forming different assemblies, namely the presence of pleomorphism within this family of enzymes. An interesting example of this behaviour has been recently shown for Cest-2923, which may exist as monomeric, dimeric and tetrameric species [24]. Although recent reports have focused on the analysis of quaternary assemblies within bacterial HSL enzymes [18], [24], a systematic classification remains to be done.

Here, we present the crystal structure of the carboxylesterase LpEst1 from *L. plantarum* WCFS1 at 2.05 Å resolution. This structure revealed that although LpEst1 belongs to the bacterial HSL family of enzymes, as otherwise expected from its sequence, it represents a novel topological variant due to the presence of large and specific structural features that decorate a canonical αβhydrolase core. Additionally, the esterase forms dimers in the crystal and in solution through an association mode not observed yet in any other αβhydrolase enzyme. Finally, *in silico* analyses of putative complexes revealed insights into the substrate specificity, which agree with the biochemical characterization of the enzyme.

## Results and Discussion

### Structure determination of LpEst1

Crystals of recombinant, His-tagged LpEst1 were prepared as previously described [25]. Analysis of the collected diffraction data revealed that these crystals were perfectly twinned, belonging to the tetragonal space group *I*4, although they exhibited apparent point group symmetry 422 [25]. Unexpectedly, some of these crystals, when manipulated, spontaneously broke up into two parts, usually yielding two equal, untwinned crystals according to intensity statistics. This phenomenon not only provided a straightforward explanation for the identified perfect twinning and the apparent 422 point group symmetry (**Figure S1**), but also opened up the possibility to prepare untwinned crystals (or crystals with a very low twin fraction) suitable for structural studies. Optimized crystallization conditions found for His-tagged, selenomethionine (Se-Met) labelled LpEst1 were 1 *M* sodium malonate, 0.5% (v/v) Jeffamine ED-2001, 100 m*M* HEPES, pH 7.0, and 5 m*M* DTT (2 μl of protein 9 mg ml^−1^ plus 2 μl of reservoir solution). Despite the fact that both native and Se-Met labelled protein variants crystallized in different conditions, crystals of Se-Met LpEst1 were essentially identical to the native ones, which allowed the collection of high quality, untwinned diffraction data (**Table 1**) that has permitted the determination of the structure of LpEst1 by anomalous diffraction methods. The initial atomic model determined was then used as a molecular-replacement search model to obtain phases for a higher resolution data set (2.05 Å resolution). This high-resolution model contains four independently refined complete protein molecules (337 amino acids each) plus residues from the N-terminal TEV recognition sequence (4 in chains A, B and D, and 2 in chain C). Also, a total of 2196 water molecules, 2 malonate molecules and 5 glycerol molecules were modelled. The final refined model has an R_work_ of 12.2% and an R_free_ of 14.2%. The average *B* factor of the structure is 30.3 Å^2^. The analysis of Ramachandran plot showed that most of the modelled residues were in preferred and allowed regions (**Table 1**).

**Table 1:** Data-collection and refinement statistics.

	Native LpEst1	SAD	High resolution
PDB code	4c88	4c87	4c89
Beamline	ID14-4 (ESRF)	ID29 (ESRF)	ID14-4 (ESRF)
Crystal parameters			
Space group	*I*4	*I*4	*I*4
Unit-cell parameters (Å)			
*a = b*	168.34	169.28	168.79
*c*	184.20	184.75	184.57
Matthews coefficient (Å^3^ Da^−1^)	4.39	4.44	4.41
Solvent content (%)	72.0	72.3	72.1
**Data-collection statistics**			
Wavelength (Å)	0.94000	0.97915	0.97914
Resolution (Å)	53.68–2.65	47.77–2.65	53.82–2.05
	(2.79–2.65)^a^	(2.79–2.65)	(2.16–2.05)
Total reflections	615388	1047518	1207617
Unique reflections	74256 (10842)	75273 (10961)	161172 (23522)
*R*_merge_	0.158 (0.508)	0.111 (0.588)	0.143 (0.702)
Mean *I/σ*	12.1 (4.4)	25.8 (4.9)	10.1 (2.9)
Completeness (%)	100.0 (100.0)	100.0 (100.0)	100.0 (100.0)
Anom. completeness (%)	-	100.0 (100.0)	-
Redundancy	8.3 (8.3)	13.9 (13.1)	7.5 (7.5)
Anom. redundancy (%)	-	7.0 (6.6)	-
**Refinement statistics**			
Protein molecules per ASU	4	4	4
Residues	1352	1356	1365
Waters	1712	823	2196
Glycerols	-	17	5
Malonate	-	-	2
Total No. of atoms	12192	11502	12839
R_work_/R_free_ (%)	13.71/17.49	14.91/18.25	12.2/14.2
Average *B* factors (Å^2^)			
All atoms	27.5	23.8	30.3
Protein	25.4	22.9	27.0
Waters	40.3	29.7	45.9
Ligands	-	66.6	54.1
R.m.s.d. from ideality			
Bonds (Å)	0.006	0.007	0.006
Angles (°)	0.960	1.049	1.025
Ramachandran plot statistics			
Preferred regions (%)	97.01	97.80	97.52
Allowed regions (%)	1.99	2.12	2.26
Outliers	0.00	0.07	0.22

a Values in parenthesis are for the outermost resolution shell.

### LpEst1 is a member of the bacterial HSL family

**Figure 1:**
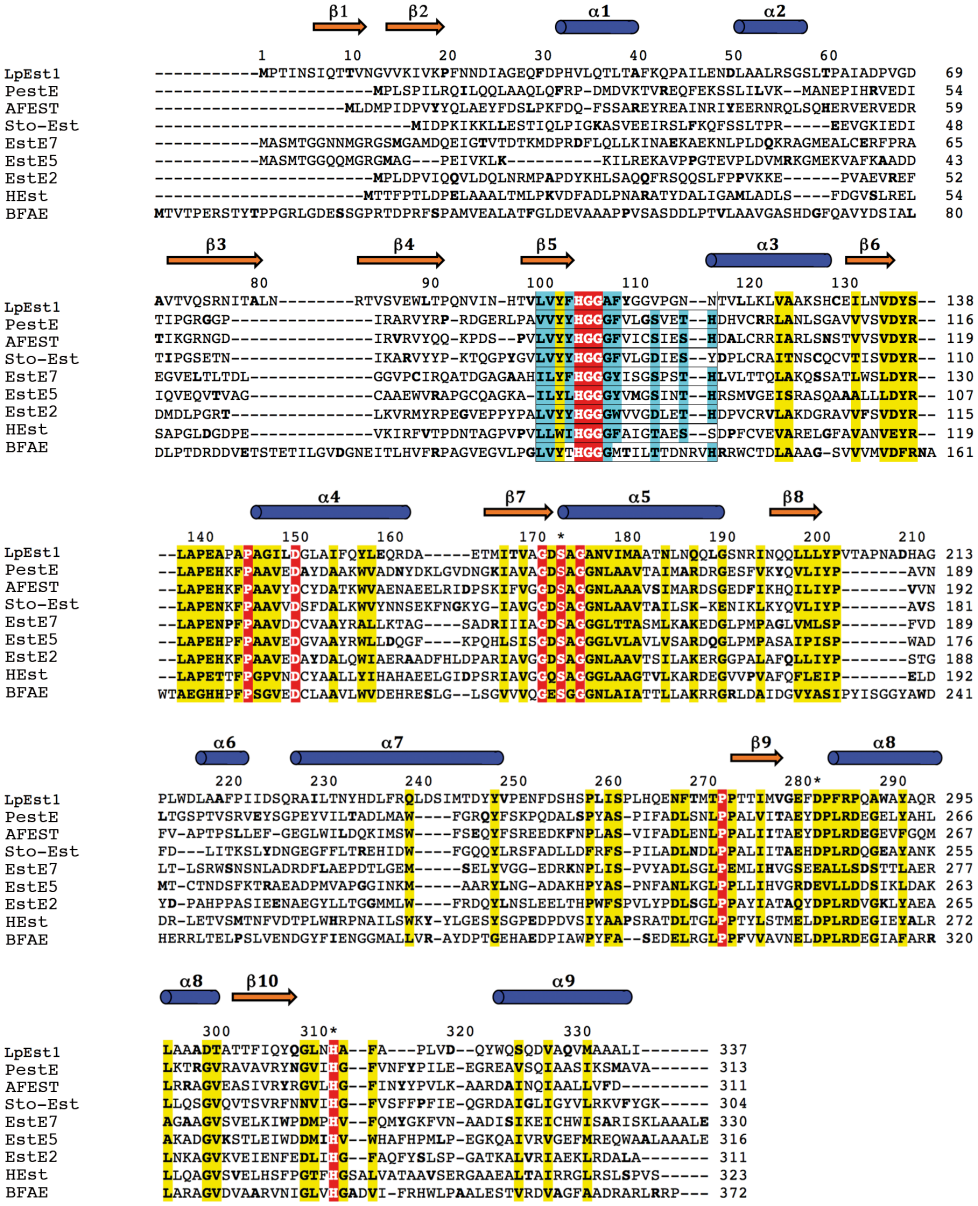
Protein sequence alignment between LpEst1 and homologs from the hormone-sensitive lipase (HSL) family. PestE, carboxylesterase PestE from *Pyrobaculum calidifontis*; AFEST, carboxylesterase AFEST from *Archaeoglobus fulgidus*; Sto-Est, thermophilic esterase Sto-Est from *Sulfolobus tokodaii*; EstE7, esterase EstE7 from environmental samples; EstE5, esterase EstE5 from environmental samples; EstE2, thermophilic esterases EstE2 from *Alicyclobacillus acidocaldarius*; HEst, heroin esterase from *Rhodococcus* sp.; BFAE, Brefeldin A esterase from *Bacillus subtilis*. Residues from LpEst1 in Residues from LpEst1 in sterase f*blue cylinders* and *orange arrows*, respectively. Residues forming the catalytic triad are marked with an asterisk. Colour code for boxes is as follows: *red*, conserved residues in all proteins; *yellow*, highly conserved positions; *cyan*, residues that coincide with the expected canonical sequence motif characteristic of enzymes from the HSL family.

The globular structure of LpEst1 subunits has approximate dimensions of 45 Å×45 Å×50 Å, and the overall secondary structure is a mixture of β-sheets (20%) and α-helices (29%). The protein belongs to the αβ-hydrolase superfamily [14] and is related to the bacterial hormone-sensitive lipase (HSL) family of enzymes [12] as concluded from structural similarity searches with DALI [26]. In this sense, multiple amino acid sequence alignment with CLUSTALW (**Figure 1**) reveals the presence in LpEst1 of the sequence motif HGGG(A) that is known to contribute to the oxyanion hole [21]. As shown in **Figure 1** this motif is localized within a sequence stretch highly conserved in the bacterial HSL enzymes, which encompasses part of strand β5 and the connecting loop between this strand and helix α3. The homologs with the highest structural similarity (**Table 2**) are hyperthermophilic carboxylesterases, namely the carboxylesterases PestE and AFEST from the archaea *Pyrobaculum calidifontis* [18] and *Archaeoglobus fulgidus* [19], respectively, followed by the mesophilic carboxylesterases from environmental samples EstE7 (PDB entry: 3k6k) and EstE5 [20], and the thermophilic esterases EstE2 and Sto-Est from *Alicyclobacillus acidocaldarius* [22] and *Sulfolobus tokodaii* [23], respectively. Additionally, high structural similarity is also found with heroin esterase from *Rhodococcus* sp. [5] and Brefeldin A esterase (BFAE) from *Bacillus subtilis* [6]. Despite a low level of average sequence identity between LpEst1 and its homologs (∼24%), the 3D structural similarity within this set of proteins is high (overall rmsd 2.3 Å for ∼280 Cα aligned atoms). This is mainly due to the presence of a common core β-sheet surrounded by α-helices, which defines the canonical αβ-hydrolase fold, which in turn typifies esterases with a Ser/Cys-His-Asp/Glu catalytic triad. On the contrary, high structural variability is observed in the so-called cap region situated over the active site on the carboxy-edge of the core β-sheet (see below), specific for the bacterial HSL family of enzymes.

**Table 2:** Structural homologs of LpEst1 as revealed by DALI.

	PDB ID	Z score	rmsd (Å)	Identity (%)	NALI^a^	NRES^b^
PestE	2YH2	36.0	2.1	22	289	308
AFEST	1JJI	34.6	2.5	26	290	311
EstE7	3K6K	34.4	2.4	20	285	297
EstE5	3FAK	33.9	2.5	18	286	297
EstE2	1EVQ	33.3	2.4	25	288	308
Sto-Est	3AIK	32.4	2.2	23	269	283
Heroin esterase	1LZL	32.4	2.4	26	285	317
Brefeldin A esterase	1JKM	32.0	2.4	21	291	361

a NALI: number of aligned residues;

b NRES: total number of residues.

### Overall structure of LpEst1: a new variant of the αβ hydrolase fold

**Figure 2:**
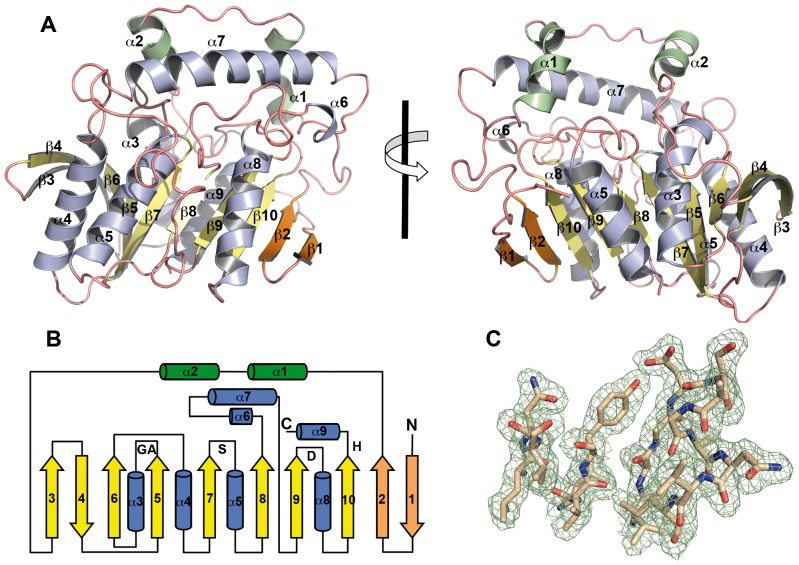
Crystal structure of the LpEst1 subunit. (A) Ribbon representation of the LpEst1 subunit; two different views are depicted. Canonical β-strands forming the core β-sheet are shown in *yellow*, whereas the two N-terminal, non-canonical ones are shown in *orange*. α-Helices are in *blue*, except the first two previous to the first β-strand from the core β-sheet (β3), which are shown in *green*. (B) Topology diagram of the LpEst1 fold. Colour code for the secondary structure elements are as in (A). The positions of the residues forming the catalytic triad Ser174, Asp283 and His313 are indicated as S, D and H, respectively, and those forming the oxyanion hole, Gly107 and Ala108, are indicated as GA. (C) Representative 2*F_obs_* – *F_calc_* density map contoured at 1σ.

LpEst1 consists of 337 residues with a molecular weight of ∼36.7 kDa (UniProtKB/Swiss-Prot code: Q88Y25). As a member of the αβ-hydrolase superfamily, the structure exhibits a three-layered architecture, namely a central, almost parallel ten-stranded β-sheet (two extra βstrands than the canonical fold) surrounded by five helices, two in the concave side of the sheet (α3 and α8) and three in the convex side (α4, α5 and α7) (**Figure 2A**). The strand order for the core β-sheet is β3, β4, β6, β5, β7, β8, β9, β10, β2 and β1 with β1 and β4 being antiparallel to the others. The remarkable finding that this arrangement, where two N-terminal strands associate to the C-terminal, outermost strand of the core β-sheet, has not been observed yet in any other αβ-hydrolase enzyme confers a novelty to the molecular topology of the bacterial HSL family (**Figure 2B**). The overall quality of the 2*F*_obs_ - *F*_calc_ electron density map can be seen in **Figure 2C**.

Together with this β-sheet sandwiched by layers of helices, members of the bacterial HSL family show an additional, mainly helical cap domain [6], [19], [22]. The cap domain of LpEst1 is formed by two sequence regions (residues 30–70 and 202–273), that group together around the carboxy-edge of the central β-sheet. Here, three α-helices (α1, α2, and α7) and irregular loops are identified. Helix α7, situated between strands β8 and β9, is exceptionally long when compared to the equivalent ones from the rest of the bacterial HSL members (see below) and mainly interacts with the extended α1–α2 connecting loop, which lies almost parallel along its axis. This helix, together with helices α1 and α2, form the upper walls of a funnel-like structure, which has the catalytic machinery at its base, with a surface area of 683 Å^2^ as determined with the CASTp server [27]. Conversely, the lower walls of this funnel are contributed by the loops between strand β5 and helix α3 and between helix α8 and strand β10. Among the residues facing the funnel ∼70% are hydrophobic, indicating the predominance of hydrophobic interactions in this environment.

**Figure 3:**
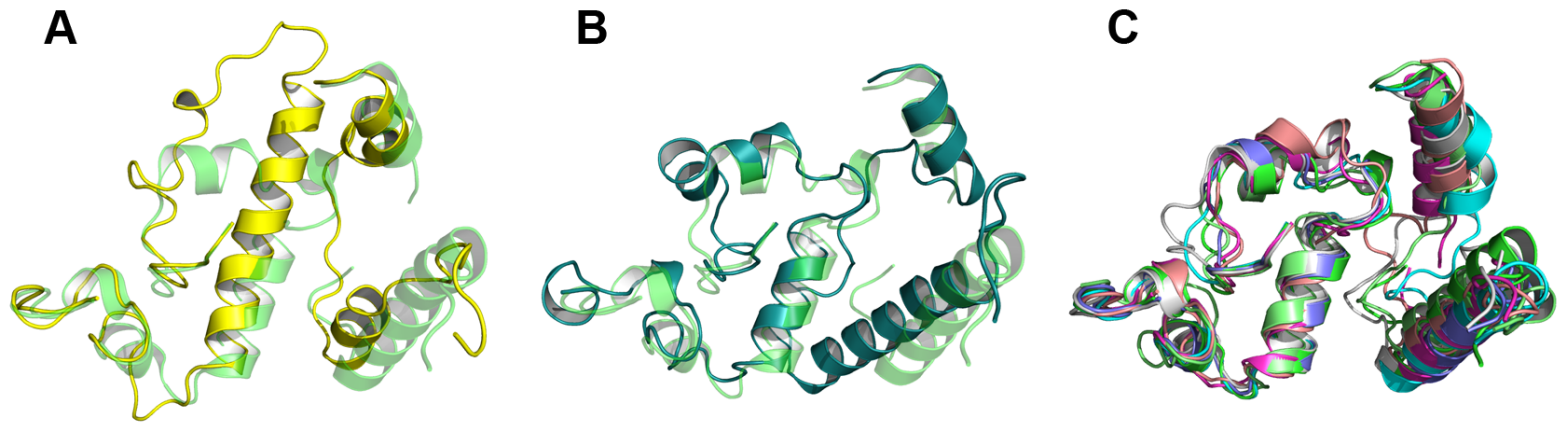
Three-dimensional comparisons of cap regions from bacterial HSL enzymes. (A) Superposition of the cap regions of LpEst1 (*yellow*) and PestE from *P. calidifontis (green*), which has been chosen arbitrarily as a representative model of the predominant pattern (see the text). (B) Superposition of the cap region from BFAE (*dark green*) and that from PestE. (C) Superposition of cap regions from PestE (*green*), AFEST (*cyan*), EstE2 (*grey*), EstE5 (*light brown*), EstE7 (*magenta*), heroin esterase (*pale green*) and Sto-Est from (*blue*). All structures are represented as ribbon models.

Structural comparisons of the cap regions of LpEst1 and its bacterial HSL homologs reveal high structural variability (**Figure 3**). Nonetheless, despite this variability three distinct patterns can be easily identified, which are represented by LpEst1 (**Figure 3A**) BFAE (**Figure 3B**) and the rest of the HSL enzymes (**Figure 3C**), respectively. This latter, predominant pattern reveals that the main source of structural variability resides in the different lengths and relative orientations of N-terminal helices (equivalent to helices α1 and α2 from LpEst1) and the corresponding connecting loop. In contrast, the C-terminal part of the cap region of these enzymes (equivalent to residues 200–273 from LpEst1) remains highly conserved (overall rmsd 1.73 Å), even in BFAE whose the N-terminal part clearly departures from the predominant pattern due to the relative displacement of helix α2 and the much longer α1–α2 connecting loop. Conversely, the cap region of LpEst1 differs from the main pattern both in the N- and C-terminal parts due to the different relative orientation of helices α1and α2 and the much longer helix α7 (22 residues versus an average of 10 residues for the rest of the proteins).

### Dimer arrangement

**Figure 4:**
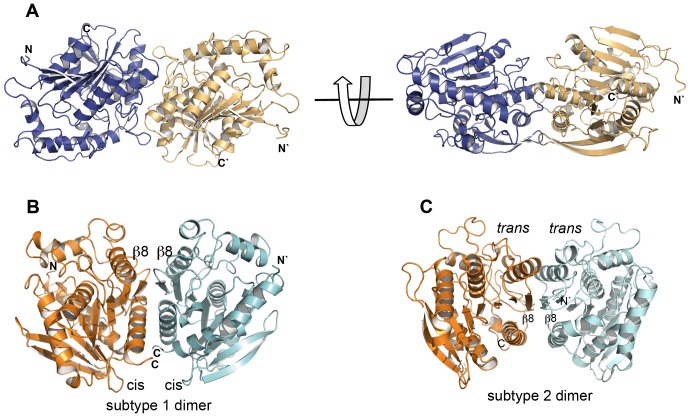
Dimeric assembly of LpEst1 and classification of the dimers of the HSL family members. (A) Two orthogonal views of a dimer of LpEst1. Each subunit is shown as ribbon model with different colour. (B) Subtype 1 of dimers (PDB entry: 3aik), characterized by a *cis-cis* association of subunits. *Cis* is arbitrarily defined as the side of the protein with respect to the plane of the core β-sheet where the α-helix downstream the canonical strand β8 is situated (see the text for details). (C) Subtype 2 of dimers (PDB entry: 1lzl), characterized by a *trans-trans* association of subunits. *Trans* is arbitrarily defined as the side of the protein with respect to the plane of the core β-sheet where the α-helix upstream the canonical strand β8 is situated. The orientation of is this dimer is as in (B).

The four independent molecules of the LpEst1 asymmetric unit are arranged in two identical dimers, consisting of the pairs A–C and B–D. The independent molecules are almost perfectly superimposable with an overall rmsd value of 0.42 Å as determined with PDBeFOLD [28]. According to the analyses with PISA [29] and PIC [30] servers, the subunits within each dimer generate an interface area of 1130 Å^2^ and interact *via* 19 hydrogen bonds and 24 hydrophobic interactions. The contacting interface is basically made up of amino acid side chains from the long connecting loops between helix α6 and strandβ9 and between helix α5 and strandβ8, and also from residues contributed by helices α4and α5 from each subunit (**Figure 4A**). It is remarkable that this region of association between LpEst1 subunits, and therefore its association mode, is different from those observed for the dimeric assemblies of the members of the HSL family, which involves the antiparallel association of the C-terminal, outermost βstrand from the core β-sheet (herein referred as to the canonical strand β8) (**Figures 4B and 4C**), as well as interactions between additional structural elements, as indicated below. Indeed, this is the case for the LpEst1 homologs, which are all (at least) dimeric species with the exception of the monomeric carboxylesterases EstE2 [22] and EstE5 [20]. Dimers adopting the above described general topological arrangement are herein defined as canonical ones. A detailed structural analysis of these canonical dimers reveals an underlying complexity, partially advanced before [18], that points to the existence of at least two distinct subtypes of dimers: *subtype* 1 canonical dimers, formed by PestE, AFEST, Sto-Est, and Brefeldin A esterase, and *subtype* 2 canonical dimers, formed by Est7 and heroin esterase. In *subtype* 1 dimers, together with the antiparallel association of the canonical strands β8 (mainly through their C-terminal ends), a large dimerization interface is made up of amino acid side chains contributed by the C-terminal α-helix from each subunit. In particular, interactions are primarily observed between the two participant C-terminal α-helices (mainly between the central part and C-terminal end of each helix) and also between the N-terminal end of these same a-helices and the N-terminal end of the canonical strand β8 from the other subunit (**Figure 4B**). Conversely, in *subtype* 2 canonical dimers the main dimerization interface is formed by amino acid side chains from the canonical strands β8 and the immediately upstream α-helices. In this case, the interactions are mainly established between equivalent structural elements from each subunit, that is, between the C-terminal, canonical β-strands themselves and the participant α-helices (**Figure 4C**). A topological corollary that can be inferred from this structural classification is that each dimer subtype is unambiguously defined by the side of the subunits (with respect to the plane of the central β-sheet) that participates in dimer formation together with the antiparallel association of the strands β8. If these sides were arbitrarily defined as *cis* and *trans*, respectively (being the *cis* side the one in which the α-helix after the canonical strand β8 is located), *subtype* 1 dimers can be defined as resulting from a *cis-cis* subunit association, whereas *subtype* 2 dimers would result from *trans-trans* subunit associations. It is notable that this structural classification correlates with the fact that proteins belonging to the subtype 1 of dimers form stable, higher order assemblies within the crystals, with the dimers as basic building blocks, whereas proteins from the subtype 2 do not. Thus, tetramers are observed in the four subtype 1 proteins considered (the biological assembly of Brefeldin A esterase has been assigned by the authors to be a tetramer; see PDB entry 1jkm), and even octamers in the case of AFEST. Structurally, the observed tetramers result from the association of subunits through their *trans* sides (**Figure S2**). This suggests that both *cis* and *trans* sides may be involved in dimer formation but only *trans* sides would be involved in tetramer formation. Of course, this does not necessarily exclude the possibility of *cis-cis* tetramers but the current structural information suggests that, should they exist, they are not abundant or canonical.

**Figure 5:**
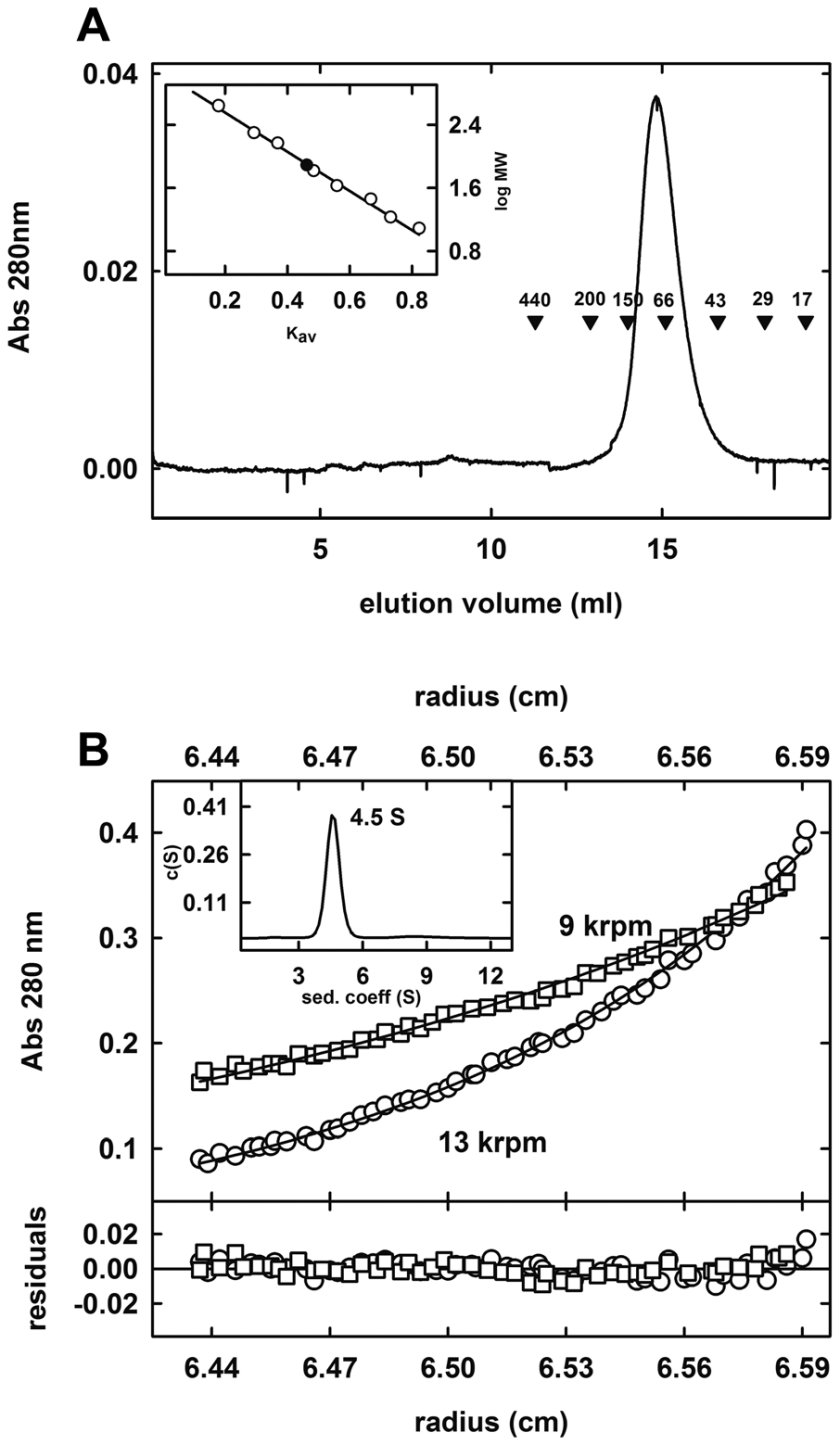
Analysis of the oligomeric state of LpEst1 in solution. (A) Analytical gel-filtration of LpEst1 on Superdex 200 10/300 GL Tricorn column. The elution profile of LpEst1 is shown together with the elution positions for some standard proteins (molecular mass in kDa). *Inset*, semilog plot of the molecular mass of all standards used *versus* their K_av_ values (*open circles*). The *closed circle* indicates the position of the K_av_ value of LpEst1 interpolated in the regression line (*solid line*) (B) Analytical ultracentrifugation analysis of LpEst1. Sedimentation equilibrium analysis of LpEst1 (10 μM) in Tris buffer (20 mM Tris-HCl, pH 8.0, and 0.1 M NaCl) at 9,000 rpm (*open* squares) and 13,000 (*open* circles). Absorbance at 280 nm is plotted against the radial position from the center of the rotor. The fit to the data set (*solid line curves*) corresponds to an ideal species with a molecular mass of 77.2±4.2 kDa (n = 3). Residuals from this fit are shown in the panel at the bottom. Calculations were done with the program Heteroanalysis [47]. *Inset*, sedimentation coefficient *c*(*s*) distributions for LpEst1 (10 μM) in Tris buffer (20 mM Tris-HCl, pH 8.0 with 0.1 M NaCl). Raw sedimentation velocity profiles for this analysis were acquired using absorbance at 280 nm, 45,000 rpm, 20 °C, and different times (not shown). Calculations were done with the program Sedfit [46].

The novelty of the association mode for LpEst1 subunits observed within the crystal prompted us to characterize the oligomeric state of LpEst1 in solution. Thus, as a first approach, we estimated the molecular weight of the enzyme by chromatographic and ultracentrifugation analyses (**Figure 5**). Results from analytical gel filtration experiments revealed that LpEst1 behaves in solution (20 mM Tris, pH 8.0, 0.1 M NaCl and 5 mM DTT) as a unique species with an apparent molecular weight of 78±3 kDa (n = 3) (**Figure 5A**), which compares well with the value theoretically expected for the dimer (77 kDa). Secondly, analytical ultracentrifugation studies revealed that LpEst1 behaves in solution as a single, homogeneous species with a sedimentation coefficient of 4.5 S (sedimentation velocity experiments) whose behaviour, as derived from sedimentation equilibrium experiments, fitted well to an ideal model of a unique species with a molecular mass of 77.2±4.2 kDa (n = 3) (**Figure 5B**). Therefore, these studies support the dimeric character of LpEst1 in solution.

### *In silico* and mutational analyses of the dimer interface

**Figure 6:**
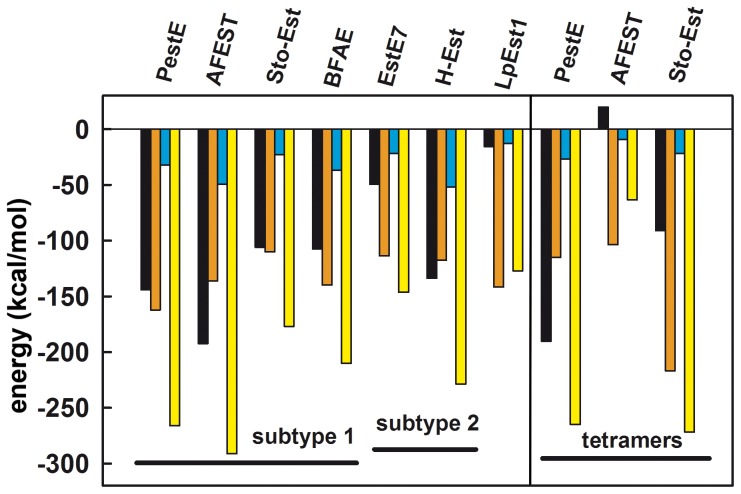
Intersubunit binding energy decomposition for dimer and tetramer formation. Colour code is as follows: *black*, electrostatic term; *orange*, van der Waals term; *blue*, hydrogen bond term; *yellow*, total contribution, including desolvation energies (not shown).

Once the dimeric nature of LpEst1 was demonstrated both in the crystal and in solution, we further analysed the contacting regions in terms of interaction energies for dimer formation of both LpEst1 and, for comparison purposes, also of its canonical homologs (**Figure 6**). Some important conclusions can be derived from this analysis: first, LpEst1 displays a low dimer stabilization energy relative to the rest of the dimeric proteins with most of this stabilization originating from apolar contacts, together with some highly directional hydrogen bonds; second, the carboxylesterases PestE [18] and AFEST [19] exhibit the highest stabilization, in agreement with their hyperthermophilic character, with the most important contributions arising from coulombic interactions and hydrogen bonds; third, a very high stabilization is observed for Brefeldin A esterase and heroin esterase, despite their mesophilic character; fourth, further stabilization is attained upon tetramer formation of PestE, AFEST and Sto-Est, with the main driving force for this association being hydrophobic interactions (**Figure 6**). The high stabilization for Brefeldin A esterase is probably explained by the fact that, in addition to the canonical contacting interfaces of subtype 1 dimers, there are numerous intersubunit contacts involving the large and protein-specific cap region. The explanation for the high stability of heroin esterase remains an open question.

**Figure 7:**
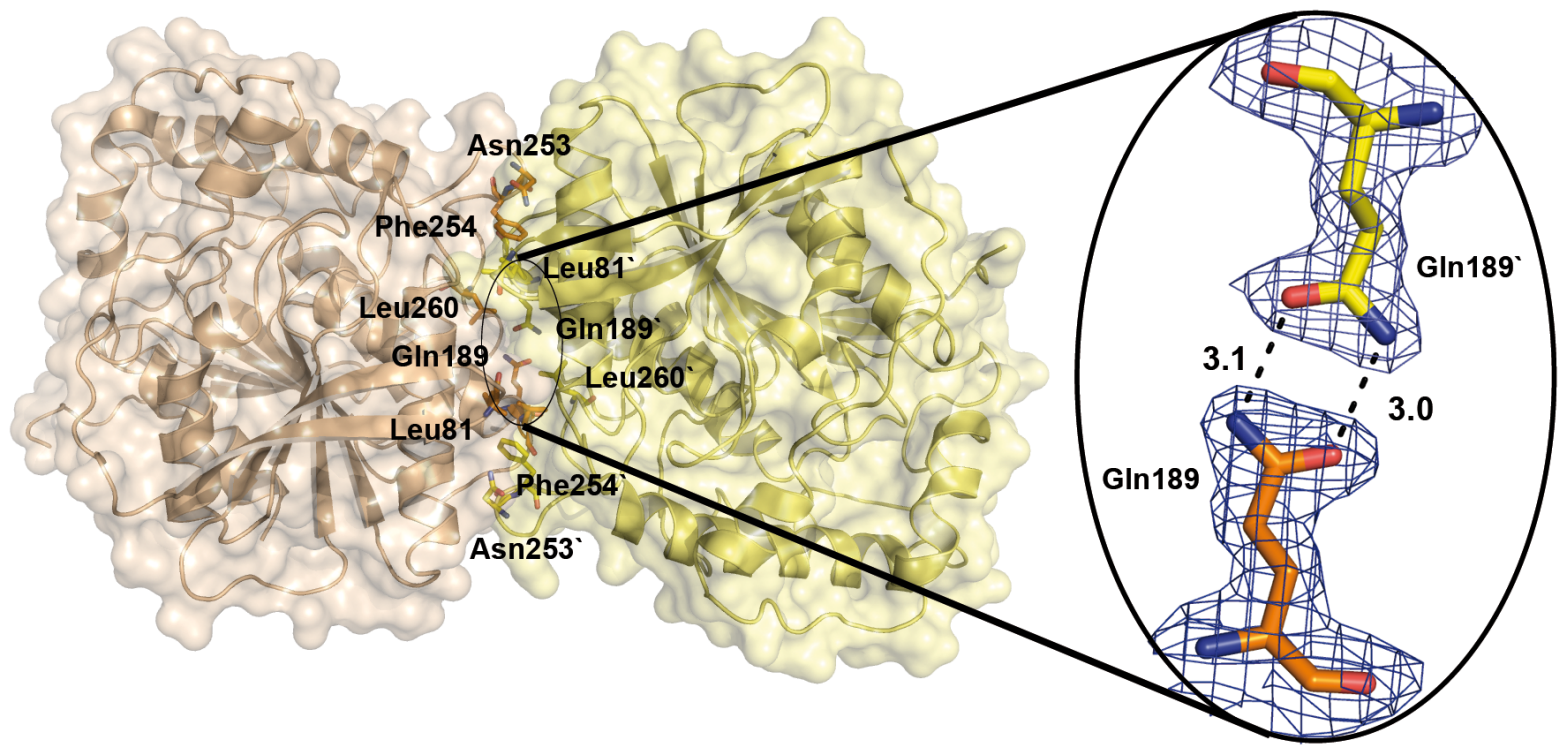
Distribution of the energetically relevant residues within the LpEst1 contacting interface. These residues are basically hydrophobic in agreement with the relevance of this type of interactions in the stabilization of the dimer. The bidentate hydrogen bonding interaction between the side-chain carboxamide groups of Gln189 (*close up view*) is situated at the core of the interface. The 2*F_obs_* – *F_calc_* density map is contoured at 1σ.

Regarding the contacting region of LpEst1 subunits, energy decomposition pinpointed six residues whose calculated contributions are larger than −6 kcal mol^−1^: Leu81, Gln189, Leu190, Asn253, Phe254 and Leu260. The fact that these residues are distributed all along the contacting interface (**Figure 7**) suggests a homogeneous stabilization in this region. Almost in the geometrical centre of the interface, a bidentate hydrogen bonding interaction is identified between the side-chain carboxamide groups of Gln189 from each subunit. Taken these characteristics into account, we raised the working hypothesis that incorporation of a charged residue at this position might destabilize this environment. With this aim, we have produced and purified the Gln189Glu mutant. We observed that after purification (IMAC on a His-Trap FF column plus size exclusion chromatography) the protein resulted metastable in solution since it slowly aggregated if maintained in 20 mM Tris–HCl, pH 8.0, 0.1 M NaCl, in contrast to the wild-type protein, which remained stable. However, when the last chromatographic step is developed under acidic conditions in McIlvaine buffer pH 5.0 (Na_2_PO_4_, citric acid, pH 5.0) no aggregation was observed for the mutant variant when stored at 4 °C for 2–3 days, which is dimeric as revealed by sedimentation equilibrium experiments in these acidic conditions (**Figure S3**). These results indicate the formation of metastable monomeric species under neutral conditions, which eventually aggregate upon the exposure of the hydrophobic interface to the bulk solvent. However, dimer formation is observed under acidic conditions due to the protonation of the Glu189 side chain. Moreover, in these acidic conditions, the Gln189Glu mutant exhibits a hydrolytic activity against *p*-nitrophenyl acetate almost equivalent to that of the wild-type enzyme.

In summary, taken as a whole, these results demonstrate that LpEst1 is a dimer despite the relative low overall interaction energies calculated at the interface and further suggest, that the apolar interactions are the main driving force for dimerization.

### Active site and substrate binding pocket

The structural homology found with enzymes from the bacterial HSL family permitted the straightforward identification of the catalytic machinery of LpEst1 as a classical catalytic triad with Ser174 as the nucleophile, His313 as the general base that deprotonates the serine hydroxyl and Asp283 as the residue that increases the pK_a_ of the histidine imidazole ring. We validated this result experimentally since the Ser174Ala, His313Ala and Asp283Ala mutants displayed no catalytic activity in terms of hydrolytic activity against *p*-nitrophenyl acetate. Significant conformational changes affecting to the global fold of the proteins as a result of the included mutations could be discarded by far-UV CD measurements (not shown).

The three catalytic residues are located at canonical positions within the αβ hydrolase fold [14]–[16], at the carboxy-edge of the core β-sheet. The nucleophile Ser174 is found at the apex of the so-called “nucleophilic elbow” between strand β7 and helix α5, within the conserved sequence motif GX_1_SX_2_G (GDSAG), a signature of the hydrolase family [14]–[16]. The constrained conformation of the nucleophile peptide backbone (φ = 56° and ψ = −128°) facilitates the formation of the “oxyanion hole” responsible for the stabilization of the negatively charged tetrahedral intermediates of the catalytic reaction [16]. Structural comparisons with other bacterial HSL homologues suggest that the oxyanion hole of LpEst1 would be formed by the backbone nitrogen atoms of Gly107, Ala108 and Ala175 namely it would be a tridentate structure similarly to those of heroin esterase [5], esterase EstE1 [31] or carboxylesterase EstE2 from *Alicyclobacillus acidocaldarius* [22]. Nonetheless, as will be shown below, the *in silico* analysis of this region discarded any role for Ala175 in the oxyanion hole.

**Figure 8:**
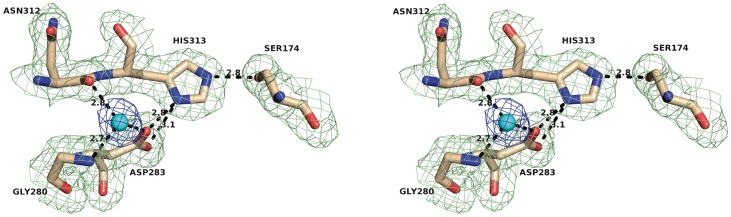
Stereoview of the network of interactions present around the catalytic triad of LpEst1. Residues forming the catalytic triad (Ser174, His313 and Asp283) and those coordinating a well-ordered water molecule that is also identified in the HSL family members (*blue sphere)* are shown as *sticks*. The 2*F_obs_* – *F_calc_* density map is contoured at 1σ (*green*: amino acid side chains; *blue*: water molecule) Distances are in Å.

A complex network of hydrogen bonds is identified in the active site (**Figure 8**). Thus, the Oγatom of Ser174 establishes a hydrogen bond (2.8 Å distance) with the Nε2 atom of the imidazole ring of His313, situated in the 13-residue loop between strand β10 and helix α9. In turn, the Nδ1 atom of this latter residue is at hydrogen bond distance to Oδ1 (3.1 Å) and Oδ2 (2.8 Å) atoms of Asp283, this residue being further stabilized by a 2.6 Å hydrogen bond with a water molecule, prototypical for the members of the HSL family [12]. This solvent molecule also forms hydrogen bonds to the carbonyl oxygen of Asn312 (2.8 Å) and the amide nitrogen of Gly280 (2.7 Å).

**Figure 9:**
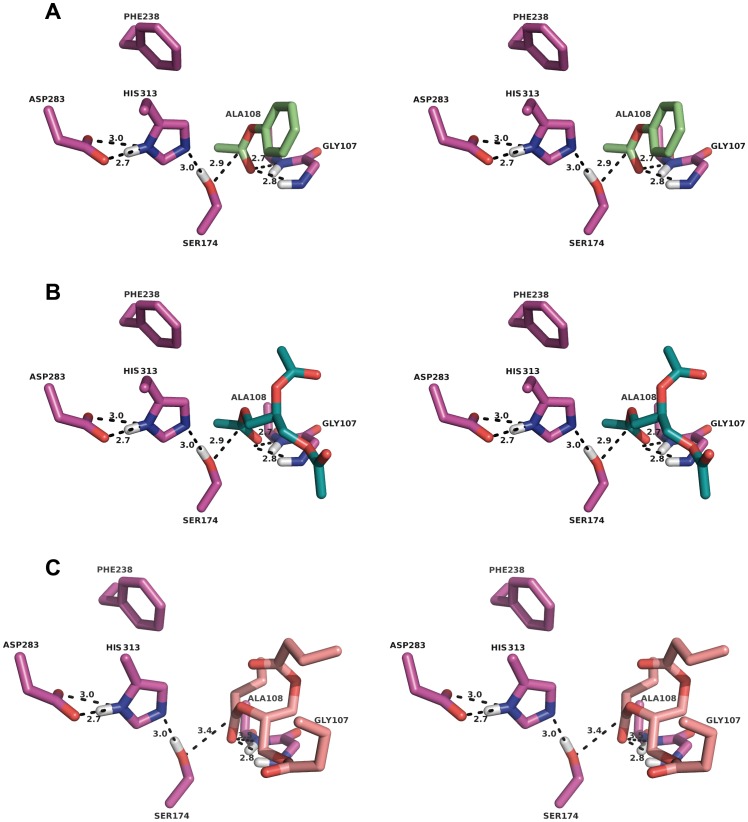
Stereoviews of three putative LpEst1:substrate complexes resulting from docking studies with CRDOCK. The three complexes correspond to phenyl acetate (a), triacetin (b) and tributyrin (c). In all cases, interactions are observed between the nucleophile (Ser174) and also reveal the stabilizing effect of the oxyanion hole formed by the backbone nitrogen atoms of Gly107 and Ala108. Residues are shown as *stick* models. Distances are in Å.

The detailed structural characterization of the LpEst1 active site permitted us to carry out docking experiments with the substrates phenyl acetate, triacetin and tributyrin (see below Biochemical characterization) (**Figure 9**). The best poses obtained from the automated docking protocol were consistent with the expected orientation of an ester-containing substrate in an esterase active site: the carbonyl moiety of the ester group is located near the catalytic hydroxyl group from Ser174 and is basically stabilized by two hydrogen bonds from the NH groups of Gly107 and Ala108, which would then form the oxyanion hole. This result rules out the backbone nitrogen atom of Ala175 as a participant in the oxyanion hole, which would thus be bidentate [16]. In addition, these three enzyme:substrate complexes reveal that the acid moiety of the substrates is inserted into a small, hydrophobic subpocket (S1) lined by Met245, Phe285, Leu241 and Val204, and the alcohol part would lie in a open and large subpocket (S2). Apparently, this particular architecture of the substrate binding-pocket in two distinct subpockets points to the hydrolytic activity of LpEst1 being directed against esters with small acid moieties but larger alcohol moieties. The analysis of substrate specificity correlates well with this prediction (see below).

**Figure 10:**
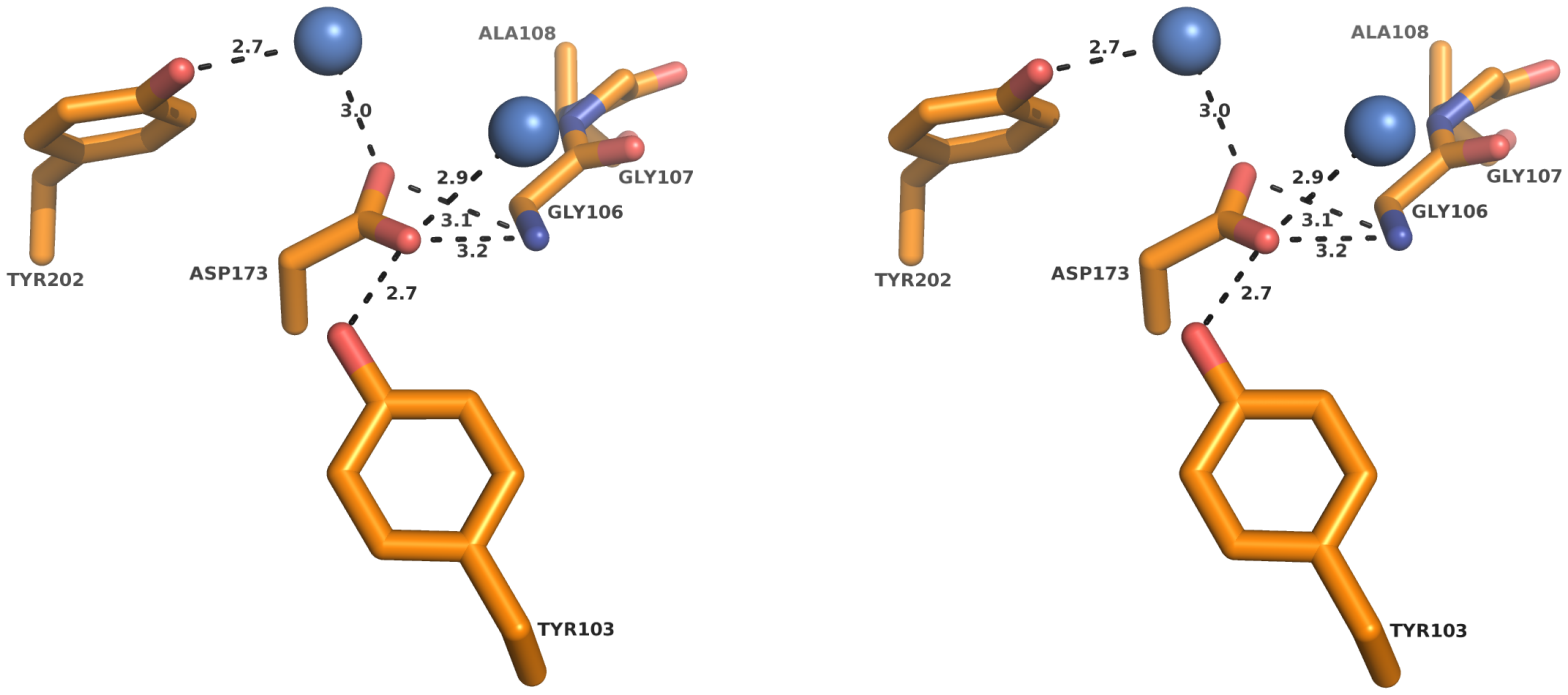
Stereoview of the environment around the residue Asp173. The side chain of the Asp173 residue is highly oriented towards the loop where the oxyanion hole resides probably contributing to its stabilization. The network of hydrogen bonds is shown as *dashed lines.* Participating residues are shown as *sticks* and water molecules as *blue spheres*. Distances are in Å.

A notable feature of the surroundings of the nucleophile Ser174 is the presence of the acidic residue Asp173. This is unexpected considering the hydrophobic character of the side chains that make up the binding pocket in LpEst1. Remarkably, all its structural homologs also have an equivalent acidic residue except for heroin esterase (**Figure 1**). In LpEst1, Asp173 participates in a network of polar interactions, some of them mediated by water molecules, and this makes the carboxylate moiety to be highly oriented towards the loop making up the oxyanion hole (**Figure 10**). Thus, the oxygen atom Oδ1 is at hydrogen bonding distance to the amide nitrogen of Gly106 (3.2 Å), the hydroxyl group of Tyr103 (2.7 Å) and a highly ordered water molecule (3.0 Å). Interestingly, the interaction between Asp173 and Tyr103 is conserved in all HSL proteins considered herein (with the exception, again, of heroin esterase). Conversely, the oxygen atom Oδ2 is at hydrogen bonding distance from the same amide nitrogen of Gly106 (3.1 Å) and a solvent water molecule (3.0 Å), which in turn interacts with the hydroxyl group of Tyr202 (2.7 Å). The orientation towards the loop making up the oxyanion hole and the well-ordered character of the Asp173 side chain suggest that this residue may play an important structural role in LpEst1, and presumably in this group of enzymes, particularly in the stabilization of the proper conformation of the oxyanion hole. In agreement with this hypothesis, the replacement of this residue by Ala (Asp173Ala) resulted in a folded (**Figure S4**) but fully inactive protein variant unable to hydrolyze *p*-nitrophenyl acetate. It is obvious that the possibility that Asp173 plays a direct functional role cannot be discarded since its acidic character should contribute significantly to the negative electrostatic potential around the active site, a feature displayed by lipases and esterases in the pH range associated with their maximum activity [2]. This aspect is currently under investigation.

### Biochemical characterization

**Figure 11:**
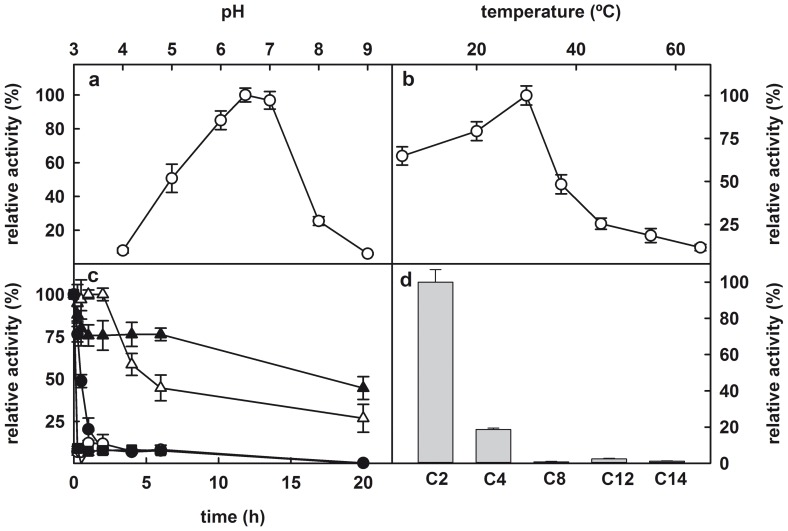
Biochemical characterization of LpEst1. (A) Dependence on pH of hydrolytic activity of LpEst1 against pNPA. (B) Dependence on temperature of hydrolytic activity of LpEst1 against pNPA. The optimum temperature for esterase activity was ∼30 °C. (C) Analysis of the temperature stability of LpEst1. The enzyme was incubated in 50 mM sodium phosphate buffer pH 7.0 at 22 °C (closed triangles), 30 °C (open triangles), 37 °C (closed circles), 45 °C (open circles) and 55 °C (closed squares) for 15 min, 30 min, and 1, 2, 4, 6 and 20 h. The values correspond to the mean of three independent experiments. (D) Dependence of the esterase activity of LpEst1 on the chain length of *p*-nitrophenyl (*p*-NP): *p*-NP acetate (C2), *p*-NP butyrate (C4); *p*-NP caprylate (C8); *p*-NP laureate (C12); and *p*-NP myristate (C14).

We have examined some important enzymatic properties of LpEst1 (**Figure 11**). The optimum pH for hydrolytic activity against *p*-nitrophenyl acetate is 6.5 (**Figure 11A**), which is a value typically observed for esterases, in contrast to the higher pH values (∼8.0) displayed by lipases [2]. Regarding to temperature, the protein presented highest activity at ∼30 °C, although at 37 °C exhibited a high level of activity (∼50%) (**Figure 11B**). These values for optimum pH and temperature are commonly found in other esterases from Lactobacilli [32]–[34]. On the other hand, temperature stability measurements show a drastic reduction in LpEst1 hydrolytic activity upon incubation of the esterase at 37 °C (**Figure 11C**).

The acyl-length selectivity against *p*-nitrophenyl ester substrates follows this order: C2>C4>C8>C12>C14, indicating a preference for short acyl-length esters (**Figure 11D**). The kinetic parameters for C2 and C4 substrates were determined spectrophotometrically. In both cases, LpEst1 exhibited a hyperbolic Michaelis-Menten kinetics (not shown). The kinetic parameters are shown in **Table 3**. From the values of these parameters it can be deduced that the catalytic efficiency (k_cat_/*K*_m_) for pNPA hydrolysis is around 8-fold the one observed for pNPB hydrolysis. Conversely, the study of the substrate profile has been qualitatively analysed with the use of a library of esters as described previously [35]. This study reveals maximum hydrolysis against phenyl acetate, and significant activity against triacetin, tributyrin and isopropenyl acetate (**Figure S5**). Apparently, this activity profile for LpEst1 basically suggests a preference for a small acid moiety of the substrate, e.g. acetate or butyrate, in good agreement with the close configuration of subpocket S1. Conversely, the absence of hydrolytic activity against other esters such as ethyl acetate, vinyl acetate and propyl acetate, for instance, clearly indicates an important influence on hydrolytic activity of the alcohol moiety of the ester. In this regard, the combination of the relaxed, open conformation of subpocket S2 with the absence of hydrolytic activity against esters with small alcohol moieties point to a threshold in the size (or length) of this part of the substrate that would be necessary to accomplish for the formation of a productive enzyme:substrate complex. Hence, as a whole, these two aspects, namely narrow substrate profile and recognition of both the acid and alcohol moieties of the substrate, would qualify LpEst1 as a specific esterase, probably reflecting a distinct biological role of the enzyme, which in turn can be linked to its unique structural peculiarities.

**Table 3:** Kinetic parameters for pNPA and pNPB hydrolysis by LpEst1.

Substrate	V_max_ (μmol min^−1^ mg^−1^)	K_m_ (mM)	k_cat_ (s^−1^)	k_cat_/V_max_ (s^−1^ mM^−1^)
pNPA	75±4	0.38±0.05	40.4±2.0	106±12
pNPB	8±1	0.2±0.1	4.4±0.3	14.7±3.3

Enzyme activities were determined at 30 °C in 50 mM sodium phosphate buffer, pH 7.0. Results are the mean value ± SD from three independent experiments.

## Materials and Methods

### DNA manipulations

The expression vector pURI3-TEV-LpEst1 coding for the wild-type esterase LpEst1 from *L. plantarum* was used as template for the preparation of single point mutants affecting to the catalytic residues, Asp173 and Gln189 residues, essentially as previously described [25], [36]: Ser174Ala, Asp283Ala and His313Ala, Asp173Ala and Gln189Glu. The mutagenic primers used (forward and reverse, respectively) were: (5′-CGTTGCTGGCGATGCGGCTGGCGC-3′) and (5′-GCGCCAGCCGCATCGCCAGCAACG-3′) for the Ser174Ala mutant, (5′-GGCGAGTTTGCTCCCTTCCG-3′) and (5′-CGGAAGGGAGCAAACTCGCC-3′) for the Asp283Ala mutant, (5′-GCTTGAACGCCGCTTTCGCACC-3′) and (5′-GGTGCGAAAGCGGCGTTCAAGC-3′) for the His313Ala mutant, (5′-GATTACCGTTGCTGGCGCTTCGGCTGGCGC-3′) and (5′-GCGCCAGCCGAAGCGCCAGCAACGGTAATC-3′) for the Asp173Ala mutant, and (5′-GCAACAAATCTGAATCAAGAACTCGGTAGC-3′) and (5′-GCTACCGAGTTCTTGAT TCAGATTTGTTGC-3′) for the Gln189Glu mutant. Production and purification of the proteins were as previously described [25].

### Overexpression and protein production

Expression and purification of Se-Met labelled LpEst1 esterase was done essentially as described for the native protein [25], using a minimum medium containing selenomethionine and buffers supplemented with 5 mM DTT. Final production yield was 6 mg per litre of culture. Incorporation of Se-Met was checked by mass spectrometry on a Finnigan LCQ Deca ion trap Mass Spectrometer (Thermo Electron, San José, CA, USA).

### Crystallization and data collection

Diffracting, perfectly twinned crystals of native LpEst1 esterase were prepared as previously described [25]. For data collection, 20% (*v/v*) glycerol was added to the crystallizing precipitant as a cryoprotectant and the crystals were flash-cooled in a 100 K nitrogen gas stream. Unexpectedly, untwinned crystals could be prepared by mechanically manipulating the first ones (see below for details). A diffraction data set was collected on ID14-4 beamline at the ESRF (Grenoble, France) using a wavelength of 0.9400 Å. An ADSC Quantum Q315r CCD detector was used with a crystal-to-detector distance of 304.72 mm, and a total of 200 images were collected with a 1° oscillation angle. The diffraction data were processed with *iMOSFLM* [37]. These untwinned crystals belong to the tetragonal space group *I*4 and have unit cell parameters a = b = 168.34 Å, c = 184.20 Å (**Table 1**). Conversely, optimized crystals of the Se-Met labelled esterase were prepared in a condition consisting of 1 *M* sodium malonate, 0.5% (*v/v*) Jeffamine ED-2001, 100 m*M* HEPES, pH 7.0, and 5 m*M* DTT (2 μl of protein 9 mg ml^−1^ plus 1 μl of reservoir solution). Similarly to the above crystals prepared with unlabelled protein, these new crystals were perfectly twinned, and their manipulation also permitted the production of untwinned crystals (or twinned crystals with a low twin fraction). The SAD data set was collected on beamline ID29 at the ESRF (Grenoble, France) using a wavelength of 0.97915 Å. The detector was a Pilatus 6 M. A total of 3600 images were collected with a 0.1° oscillation angle and a crystal-to-detector distance of 388.02 mm. The diffraction data were processed with the *XDS* [38] program package. The crystal belonged to the tetragonal space group *I*4, with unit cell parameters a = b = 169.28 Å, c = 184.75 Å (**Table 1**). Interestingly, these latter crystals on average diffracted at higher resolution than those prepared with unlabelled protein. Hence, the high-resolution data set was collected with one of these crystals. This data set was collected on beamline ID14-4 at the ESRF using a wavelength of 0.97914 Å and an ADSC Quantum Q315r CCD detector. The crystal-to-detector distance was 279.47 mm, and a total of 360 images were collected with a 0.5° oscillation angle. The diffraction data set was processed with *iMOSFLM* [37]. In all cases, space group examination was done with *POINTLESS* [39] and intensity scaling and reduction with *SCALA* [39] from the *CCP4* suite of programs [40]. Analysis of data quality and merohedral twinning was done with both *TRUNCATE* [41] and *phenix.xtriage* [42].

### SAD phasing and structure solution

The structure of LpEst1 esterase was determined by single-wavelength anomalous diffraction (SAD) at the optimal peak wavelength. SAD phasing and model-building was carried out with *AutoSol* and *AutoBuild* from *PHENIX* [42], respectively. *AutoSol* could determine the complete Se substructure and an initial electron density map could be calculated. The model automatically built was made up of 1337 residues in 7 fragments. The remaining model was built manually using *Coot* [43] and the refinement was performed with *phenix.refine* [44]. The data-collection and refinement statistics are summarized in **Table 1**. The atomic coordinates and structure factors for LpEst1 have been deposited in the Protein Data Bank (PDB entry 4c87).

### Structure determination and refinement of the high-resolution data set

Phases for the native and the high-resolution data set were obtained by molecular replacement using the program *Phaser* [45]. The atomic coordinates of the SAD structure were used as a search model. As above, the model was built manually using *Coot* [40] and the refinement was performed with *phenix.refine* [44]. Refinement steps included *xyz* refinement, TLS, individual atomic displacement parameters (ADPs), addition of ligands, and automatic addition of water molecules using default parameters. The complete data-collection and refinement statistics are shown in Table 1. Since a low fraction of twinning was detected in the high-resolution data set (0.17 Britton analysis or H-test; 0.161 maximum likelihood method), the final refinement was carried out by applying the suggested twin law (-h, k, -l). Validation of the model was carried out using *MolProbity* [46]. The atomic coordinates and structure factors have been deposited in the Protein Data Bank (PDB entries 4c88 and 4c89 for native and high-resolution Se-Met labelled LpEst1, respectively).

### Figure preparation

Model representation was done with *PyMOL* [47].

### Analytical chromatography

Analytical size-exclusion chromatography was performed on a Superdex 200 10/300 GL Tricorn column (GE Healthcare) equilibrated in 20 mM Tris–HCl, pH 8.0, 0.1 M NaCl, 5 mM DTT and 0.04% (w/v) sodium azide. The column was calibrated with apoferritin (443 kDa), β-amylase (200 kDa), alcohol deshydrogenase (150 kDa), bovine serum albumin (66 kDa), ovalbumin (45 kDa), carbonic anhydrase (29 kDa), sperm whale myoglobin (17 kDa) and vitamin B_12_ (1.3 kDa) in the same buffer. The size of LpEst1 was determined from its K_av_ value (K_av_ = (V_e_−V_0_)/(V_T_−V_0_); V_e_: elution volume; V_0_: void volume; V_T_: total volume of the column) by interpolation in a calibration semilog plot of the molecular mass of the standard proteins versus their K_av_ values.

### Analytical ultracentrifugation

Equilibrium and velocity ultracentrifugation experiments were performed using a Beckman XL-A ultracentrifuge with an An-50Ti rotor and standard double sector centrepiece cells. Solvent density (1.002 mg ml^−1^) and the partial specific volume of LpEst1 (0.719) were calculated from the buffer composition (100 m*M* NaCl and 20 m*M* Tris) and from the predicted amino acid composition, respectively, with SEDNTERP [48]. Data from sedimentation velocity and equilibrium experiments were analysed with the programs Sedfit [49] and Heteroanalysis [50], respectively.

### Circular dichroism spectroscopy

Far-UV circular dichroism (CD) measurements were carried out on a Jasco J-715 spectropolarimeter equipped with a thermostated cell holder and a Peltier temperature control accessory. The instrument was calibrated with (+)-10-camphorsulfonic acid. CD spectra were recorded in 0.1 cm path length quartz cells cuvettes from 250 to 200 nm at 25 °C, using a protein concentration of 5.5 μM (1 nm bandwidth, 4 s response, and 20 nm/min scan speed). Each spectrum herein presented is the average accumulation of four scans. Baseline subtraction was performed in all cases. Results are expressed as mean residue ellipticity [θ]_MRW_, in units of degree cm^2^ dmol^−1^ of amino acid (Mr  = 110 for this protein). Thermal transitions were also analyzed by CD spectroscopy by monitoring the variation of the ellipticity at 222 nm as the temperature was increased from 20 to 90 °C at 50 °C/h. The normalized ellipticity value at each temperature was calculated as ([θ]_T_−[θ]_25_)/([θ]_90_−[θ]_25_), where [θ]_T_ is the ellipticity value at temperature T, and [θ]_25_ and [θ]_90_ are the ellipticity values at 25 °C and 90 °C, respectively. Three different samples were analysed, although the traces shown correspond to individual samples.

### Enzyme assays and biochemical characterization of LpEst1

Esterase activity was examined spectrophotometrically using *p*-nitrophenyl acetate as substrate. The rate of hydrolysis of *p*NP-acetate for 10 min at 30°C was measured in 50 mM sodium phosphate buffer pH 7.0 at 348 nm in a spectrophotometer (UVmini-1240 Shimadzu). The amount of protein used was 10 μg. The reaction was stopped by chilling on ice. Controls without enzyme were utilized to account for any spontaneous hydrolysis. One unit of esterase activity was defined as the amount of enzyme required to release 1 μmol of *p*-nitrophenol per minute under the previously described conditions.

Substrate specificity of LpEst1 was examined using different *p*-nitrophenyl (*p*-NP) esters of various chain lengths (C2, C4, C8, C12, C14 and C16) and substrate profile was analysed with a library of esters similar to the one reported previously [35]. The esters were chosen to identify acyl chain length preferences of LpEst1 and also its ability to hydrolyse hindered or charged substrates. Simple alkyl esters as well as activated esters (vinyl and phenyl esters, esters with electron-withdrawing substituents in the acyl portion) were included to test whether activated esters would react faster. The screening was performed in a 96-well plate Flat Bottom (Sarstedt) with a final reaction volume of 200 μl per well, each one containing 1 m*M* substrate in acetonitrile (1% *v/v*). The buffer/indicator solution contained 0.44 m*M* of *p*-nitrophenol as pH indicator in 1 m*M* sodium phosphate buffer pH 7.2. Esterase (20 μl of a 0.5 mg ml^−1^ solution in 1 m*M* sodium phosphate buffer pH 7.2) was added to each well and reactions were followed by measuring the decrease in absorbance at 410 nm for 2 h at 30°C in a Synergy HT BioTek microplate spectrophotometer. The incubation time was selected as to maximize the signal-to-noise ratio of the absorbance readings. Controls without enzyme carried out for each substrate indicated that compounds were stable within the time scale of the experiments. Data were collected in triplicate and the average activities were quantified. Results are shown as means ± standard deviations.

In order to investigate temperature effect, reactions were performed in 50 mM sodium phosphate buffer (pH 7.0) at 4, 20, 30, 37, 40, 45, 55 and 65 °C. Effect of pH was investigated by assaying esterase activity in a range of pH values from 5.5 to 9.0 at 30 °C. Buffers used were acetic acid-sodium acetate buffer for pH 5.5, sodium phosphate buffer for pH 6–7, Tris-HCl buffer for pH 8 and glycine-NaOH buffer for pH 9. A 100 mM concentration was used in all the buffers.

For temperature stability measurements, the recombinant esterase was incubated in 50 mM sodium phosphate buffer pH 7.0 at 20, 30, 37, 45, 55 and 65 °C for 15 min, 30 min, and 1, 2, 3, 4, 6 and 20 h. After incubation, the residual activity was measured as described above.

### Computational methods

The *pdb2pqr.py* tool [51] was used to estimate the most probable protonation states of titratable residues in the protein at a pH of 7.0 and to add all the missing hydrogen atoms. Hydrogens belonging to the catalytic triad (Ser174, His313 and Asp283) were manually reoriented in accordance with the known catalytic mechanism of the enzyme.

Affinity potentials [52] within the active site for methyl (sp^3^ C), hydroxyl (H-bond acceptor/donor sp^3^ O), carbonyl oxygen (H-bond acceptor sp^2^ O), positively charged amino (H-bond donor sp^3^ N) and hydrophobic (sp^3^ C minus a H-bonding term) probes were calculated with our in-house program cGRILL using a grid spacing of 0.5 Å. Substrates phenyl acetate, triacetin and tributyrin were built and assigned point charges and parameters from the Merck molecular force field 94 using the openbabel tool. Energetically favoured binding poses for these substrates within the active site were found by using our in-house CRDOCK tool [53]. Briefly, for each substrate a conformational search was performed and exhaustive sampling was achieved using the AMBER force field for intermolecular energy evaluation and the BFGS algorithm for pose optimization. The final binding modes selected were awarded the best score according to the CRScore function.

The nature of the interactions and the residues involved in dimerization of LpEst1 and its bacterial HSL homologs were analyzed by means of the MM-ISMSA method [54], which includes the non-bonding term of the AMBER99sb force field [55] and a desolvation term as calculated by the Implicit Solvation Method (ISM) [56]. The per-residue energy decomposition performed by this tool also allowed us to identify those amino acids that contribute to the overall stabilization of the dimer. For adaptation to the AMBER99sb force field, all proteins were first protonated at pH = 7.0 using PDB2PQR, parameterized and geometry optimized by following a simple protocol of 500 steps of steepest-descent and 4500 conjugate-gradient energy minimization with SANDER [57].

## Supporting Information

Figure S1 **Crystals of Se-Met labelled LpEst1.** (A) Crystallization drop containing both spindle-shaped crystals of Se-Met labelled LpEst1, which corresponded to perfectly twinned crystals with apparent point group 422 (apparent space group *I*422) and “half” crystals resulting from the manipulation of the latter, which belonged to the tetragonal *I*4 space group and did not exhibit merohedral twinning. Bar length corresponds to 0.2 mm. (B) Diagram explaining the perfect twinning present in the spindle-shaped crystals of LpEst1 as resulting from the geometrically well defined combination of two opposed, untwinned crystals.
(TIF)

Figure S2 **Tetramers formed by canonical** ***subtype*** **1 dimers of enzymes from the hormone-sensitive lipases family.** (A) tetramer of the thermophilic esterase St-Est from *Alicyclobacillus acidocaldarius* (PDB entry, 3aik). B, tetramer of the hyperthermophilic carboxylesterase PestE from the archaea *Pyrobaculum calidifontis* (PDB entry, 2yh2). The dimers at the bottom are oriented as in Fig. 3 and are shown as *ribbon* models, whereas the upper dimers are shown as *surface* plus *ribbon* models.
(TIF)

Figure S3 **Analytical ultracentrifugation studies of LpEst1 Gln189Glu mutant.** Sedimentation equilibrium analysis of LpEst1 (10 μM) in McIlvaine buffer pH 5.0 (Na_2_PO_4_, citric acid, pH 5.0) at 12,000 rpm (*open* squares) and 18,000 (*open* circles). Absorbance at 280 nm is plotted against the radial position from the center of the rotor. The fit to the data set (*solid line curves*) corresponds to an ideal species with a molecular mass of 77.4±2.2 kDa (n = 3). Residuals from this fit are shown in the panel at the bottom. Calculations were done with the program Heteroanalysis [47].
(TIF)

Figure S4 **Circular dichroism analysis of wild-type and LpEst1 Asp173Ala mutant.** (A) Far-UV CD spectra of wild-type LpEst1 (*open circles*) and Asp173Ala mutant (*open triangles*). Spectra were recorded in 20 mM Tris-HCl, pH 8.0, and 0.1 M NaCl. Protein concentration was 0.2 mg/ml. (B) Heat denaturation curves for LpEst1 in the same experimental conditions as in (A) (see Materials and Methods for further details).
(TIF)

Figure S5 **Analysis of the substrate specificity of LpEst1.** Activity values are normalized to the maximum value, which is observed for triacetin. *Right*, list of substrates used in the ester library.
(TIF)
