# Supplementary figures and images for: Esterase LpEst1 from Lactobacillus plantarum: A Novel and Atypical Member of the αβ Hydrolase Superfamily of Enzymes
Source: PLoS One. 2014 Mar 24;9(3):e92257. doi: 10.1371/journal.pone.0092257 (PMC3963902; doi:10.1371/journal.pone.0092257)

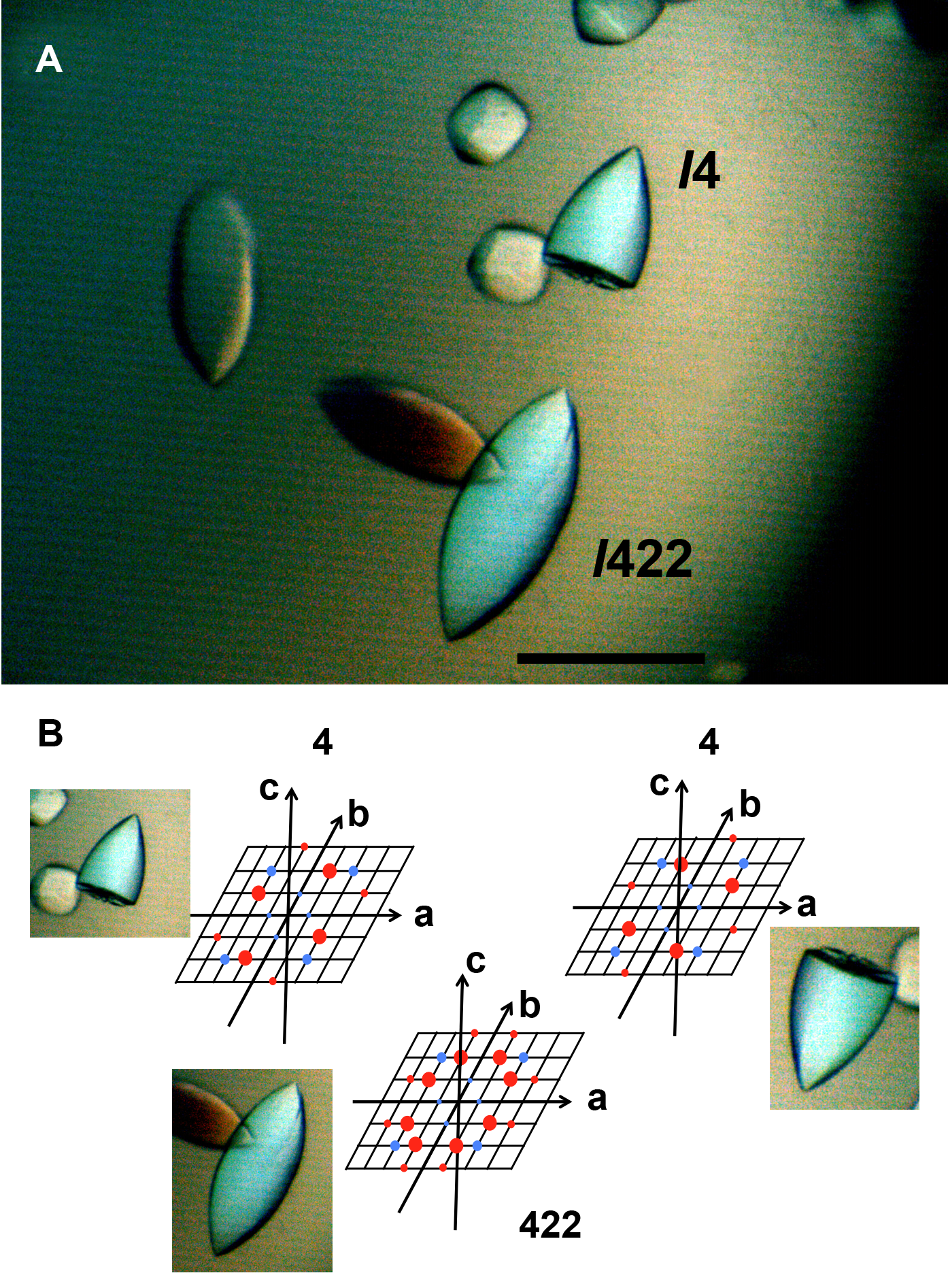

Supplement: Figure S1 — Crystals of Se-Met labelled LpEst1. (A) Crystallization drop containing both spindle-shaped crystals of Se-Met labelled LpEst1, which corresponded to perfectly twinned crystals with apparent point group 422 (apparent space group I422) and “half” crystals resulting from the manipulation of the latter, which belonged to the tetragonal I4 space group and did not exhibit merohedral twinning. Bar length corresponds to 0.2 mm. (B) Diagram explaining the perfect twinning present in the spindle-shaped crystals of LpEst1 as resulting from the geometrically well defined combination of two opposed, untwinned crystals. (TIF) [file pone.0092257.s001.tif]

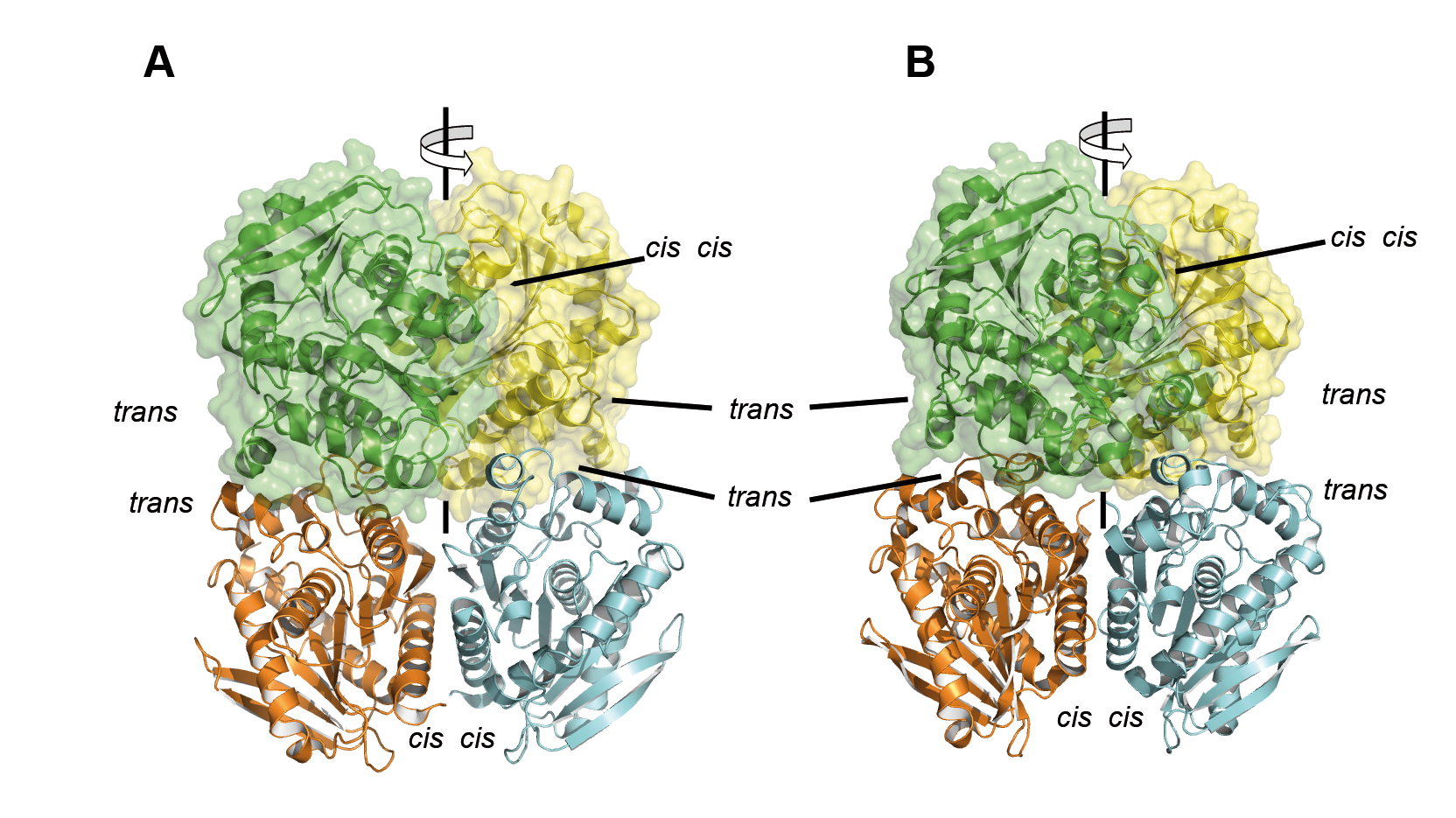

Supplement: Figure S2 — Tetramers formed by canonical subtype 1 dimers of enzymes from the hormone-sensitive lipases family. (A) tetramer of the thermophilic esterase St-Est from Alicyclobacillus acidocaldarius (PDB entry, 3aik). B, tetramer of the hyperthermophilic carboxylesterase PestE from the archaea Pyrobaculum calidifontis (PDB entry, 2yh2). The dimers at the bottom are oriented as in Fig. 3 and are shown as ribbon models, whereas the upper dimers are shown as surface plus ribbon models. (TIF) [file pone.0092257.s002.tif]

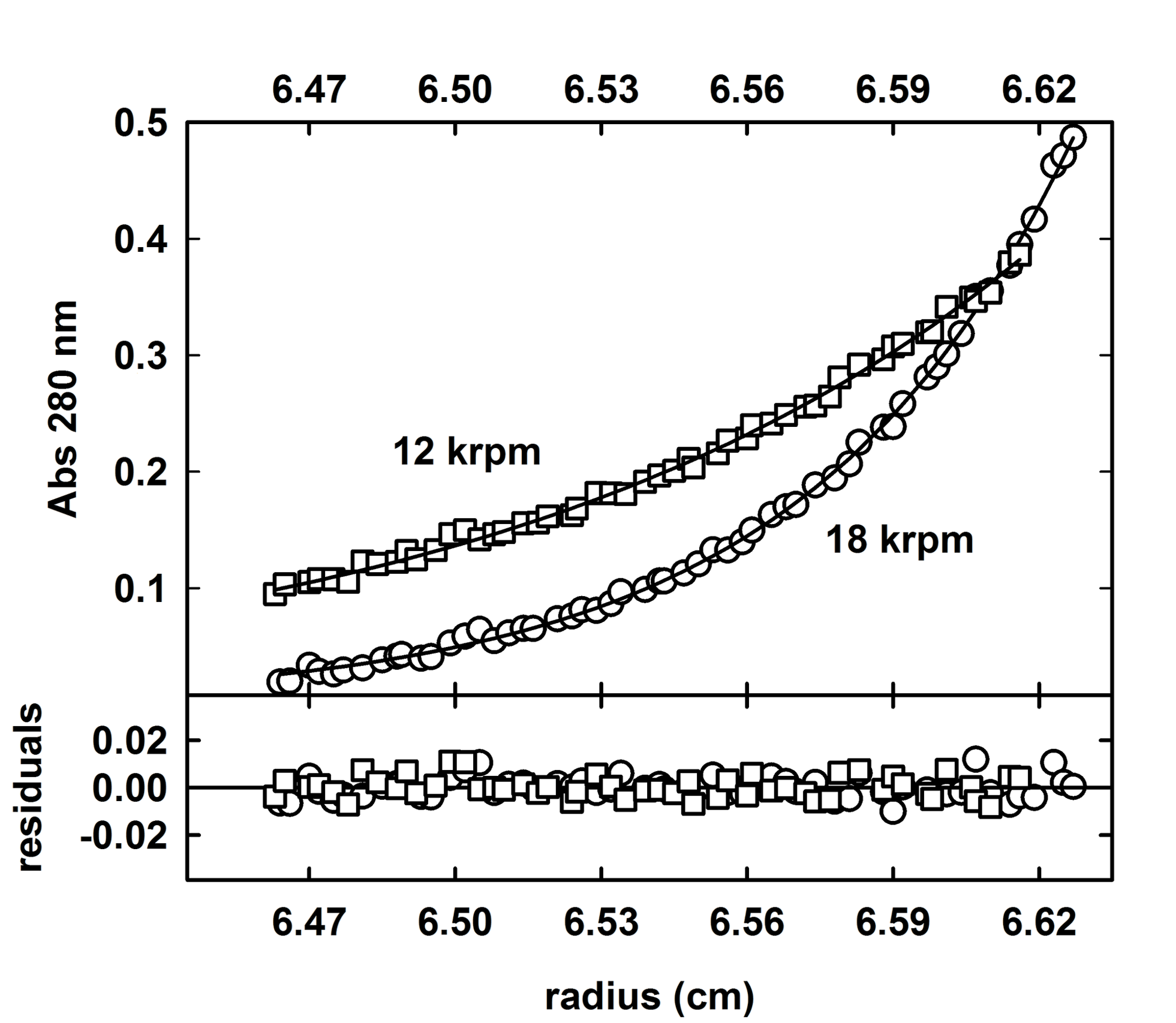

Supplement: Figure S3 — Analytical ultracentrifugation studies of LpEst1 Gln189Glu mutant. Sedimentation equilibrium analysis of LpEst1 (10 μM) in McIlvaine buffer pH 5.0 (Na2PO4, citric acid, pH 5.0) at 12,000 rpm (open squares) and 18,000 (open circles). Absorbance at 280 nm is plotted against the radial position from the center of the rotor. The fit to the data set (solid line curves) corresponds to an ideal species with a molecular mass of 77.4±2.2 kDa (n = 3). Residuals from this fit are shown in the panel at the bottom. Calculations were done with the program Heteroanalysis [47]. (TIF) [file pone.0092257.s003.tif]

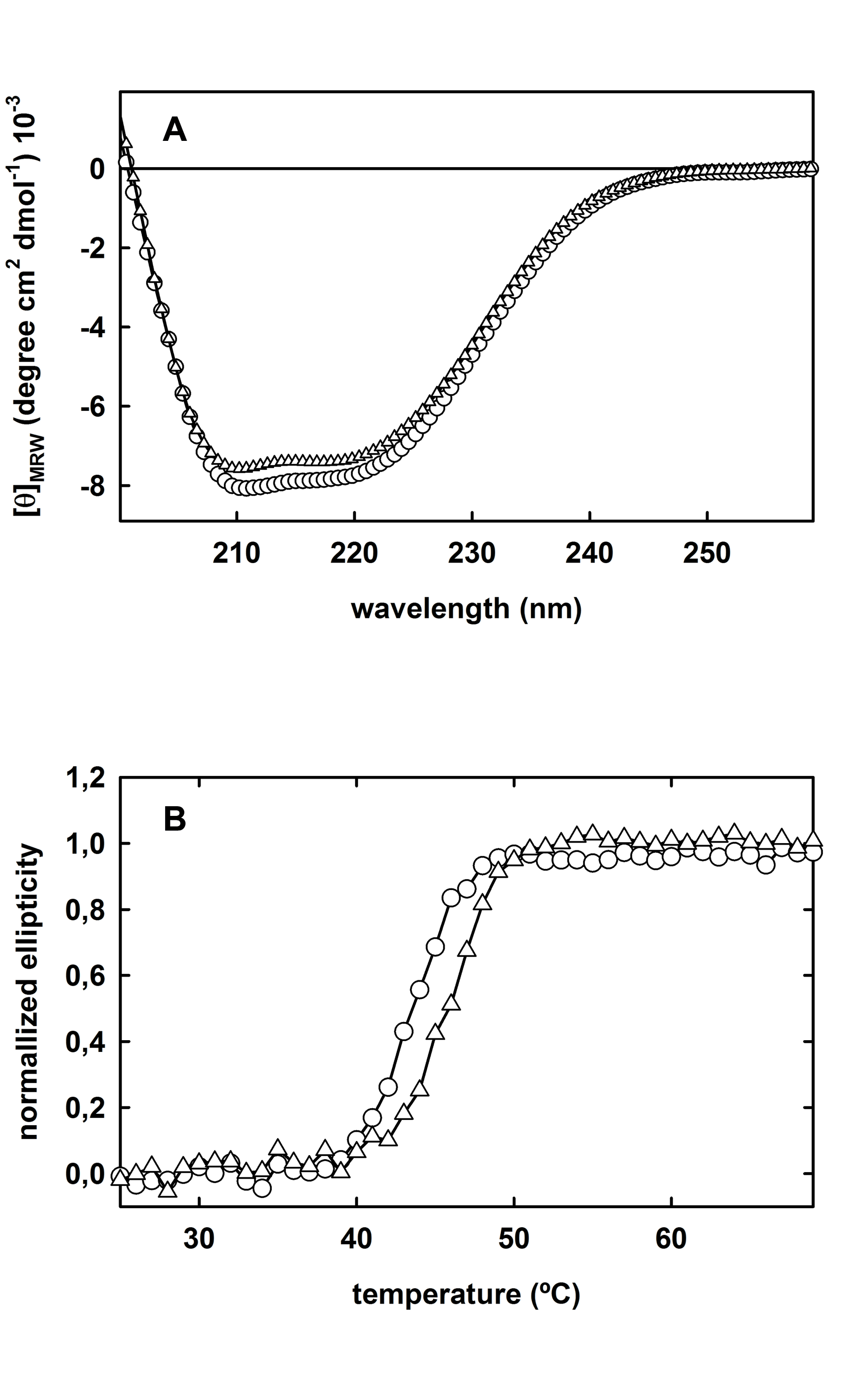

Supplement: Figure S4 — Circular dichroism analysis of wild-type and LpEst1 Asp173Ala mutant. (A) Far-UV CD spectra of wild-type LpEst1 (open circles) and Asp173Ala mutant (open triangles). Spectra were recorded in 20 mM Tris-HCl, pH 8.0, and 0.1 M NaCl. Protein concentration was 0.2 mg/ml. (B) Heat denaturation curves for LpEst1 in the same experimental conditions as in (A) (see Materials and Methods for further details). (TIF) [file pone.0092257.s004.tif]

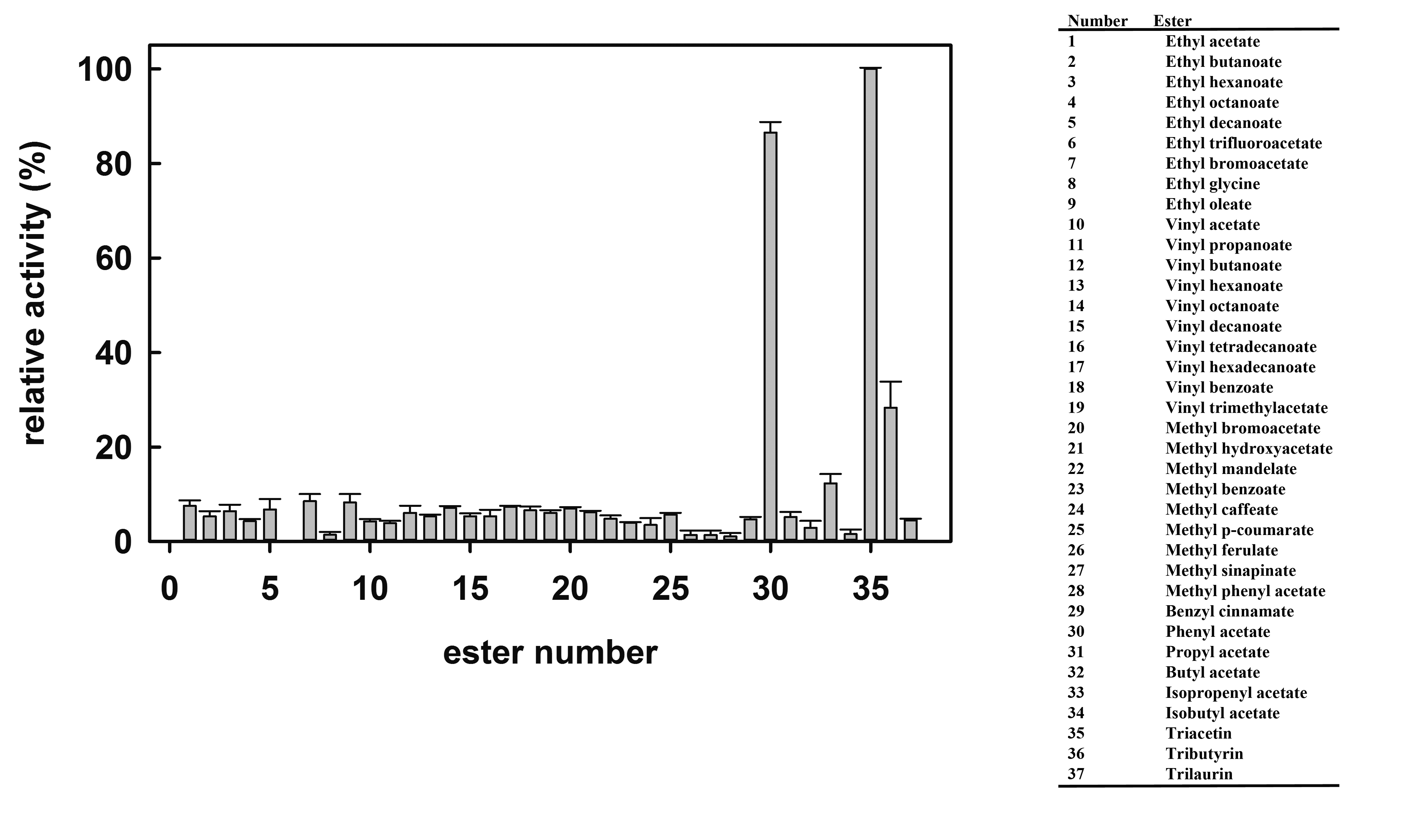

Supplement: Figure S5 — Analysis of the substrate specificity of LpEst1. Activity values are normalized to the maximum value, which is observed for triacetin. Right, list of substrates used in the ester library. (TIF) [file pone.0092257.s005.tif]
